# Prebiotic Maltose Gel Can Promote the Vaginal Microbiota From BV-Related Bacteria Dominant to *Lactobacillus* in Rhesus Macaque

**DOI:** 10.3389/fmicb.2020.594065

**Published:** 2020-11-06

**Authors:** Qiong-qiong Zhang, Zhi-heng Liu, Li-ling Liu, Gang Hu, Guang-lun Lei, Ying Wang, Yang Cao, Wei Wu, Lei Zhang, Qin-ping Liao

**Affiliations:** ^1^ School of Clinical Medicine, Tsinghua University, Beijing, China; ^2^ Department of Obstetrics and Gynecology, Beijing Tsinghua Changgung Hospital, School of Clinical Medicine, Tsinghua University, Beijing, China; ^3^ School of Life Sciences, Peking University, Beijing, China; ^4^ Shenzhen Eulikan Biotechnology Co., Ltd, Shenzhen, China; ^5^ Sichuan Green-house Biotech Co., Ltd, Sichuan, China; ^6^ Suzhou Turing Microbial Technologies Co., Ltd, Suzhou, China

**Keywords:** maltose gel, vaginal microbiota, *Lactobacillus*, bacterial vaginosis, rhesus macaque

## Abstract

The high incidence of bacterial vaginosis recurrence is common after treatment with an antibiotic agent and suggests the need for new treatments to prevent this. We conducted a randomized trial to evaluate the ability of maltose gel to treat bacterial vaginosis. Eighteen female rhesus macaques were randomly assigned, in a 2:1 ratio, to receive maltose gel or placebo gel by syringe to the fornix of the vagina for five consecutive days. We used 16S rRNA sequencing data from 70 swab samples of vaginal secretions in two groups in total on days 0, 3, and 5 after medication initiation and days 3 and 5 after medication withdrawal for the study of microbiome composition. We found that, in the placebo control group, there was no significant change in the composition and abundance of vaginal microbiota during the follow-up period. In the maltose gel test group, the abundance of *Lactobacillus* in the vagina microbiota increased gradually with the prolongation of the treatment time on Days 3 and 5 (ANOVA *p* = 6.99e−5 < 0.01) but began to decrease after the withdrawal of maltose gel, which was different from that of the control group. Correspondingly, the diversity and abundance of BV-related bacteria, *Fusobacterium*, *Parvimonas*, *Mobiluncus*, *Campylobacter*, *Prevotella*, and *Sneathia*, decreased on Day 0 to Day 5 of medication and increased after drug withdrawal in the maltose gel test group. The study confirms that maltose gel can facilitate the proliferation of *Lactobacillus* and promote the transition of the vaginal microbiota from BV-related bacteria dominant to *Lactobacillus* dominant in the rhesus macaque.

## Introduction

Bacterial vaginosis (BV) is the most common lower genital tract disorder of fertile women (Mendling et al., 2019) and has been associated with an increased risk of adverse pregnancy outcomes (Leitich and Kiss, 2007; Klebanoff and Brotman, 2018), pelvic inflammatory disease (Taylor et al., 2013), and various sexually transmitted illnesses (STIs; Atashili et al., 2008). Metronidazole and clindamycin are the optimal choices for the treatment of BV worldwide. Although antibiotics represent an effective therapeutic option in the short term, recurrence remains a serious problem (Russo et al., 2019; Cohen et al., 2020). The high incidence of relapses indicates that new agents are needed to treat BV.

*Lactobacillus*, the most prevalent microbiota in the vagina, protect it from pathogenic infections by producing antimicrobial compounds (e.g., hydrogen peroxide, lactic acid, and bacteriocin-like substances) and by adhering and competing for adhesion sites in the vagina (Borges et al., 2014; Yefet et al., 2020). BV is characterized by shifts in the vaginal microbiota from *Lactobacillus* dominant to a microbiota with diverse anaerobic bacteria (Srinivasan et al., 2015). In recent years, the use of micro-ecological preparations to restore the dominant position of *Lactobacillus* has become a new strategy for the treatment and prevention of BV recurrence (Gaziano et al., 2020). A previous study found that individuals with *Lactobacillus crispatus*-dominant vagina microbiome communities show extremely lower risks of acquiring human immunodeficiency virus (HIV) when compared with individuals with diverse vagina bacterial communities dominated by anaerobes (Gosmann et al., 2017). Recent research also found that *Lactobacillus*-dominant vagina microbiome shows a protective effect against the human papillomavirus (HPV) infection, and cervical intraepithelial neoplasia (CIN), which is considered to be a precursor of cervical carcinoma (Yang et al., 2018; Mitra et al., 2020).

Prebiotic refers to a food ingredient that cannot be further digested and has a beneficial effect on the host by selectively promoting the growth and/or enhancing the activity of one or several bacteria (Gibson and Roberfroid, 1995). Artificially produced prebiotics includes lactulose, galactooligosaccharides, fructooligosaccharides, maltooligosaccharides, cyclodextrins, lactosaccharose, etc. (Markowiak and Slizewska, 2017). α-Amylases were found in women’s vagina fluid, which can break down glycogen into maltose, maltotriose, and maltotetraose, which are important to support the growth of *Lactobacillus* (Spear et al., 2014). Though, under low pH environment, α-amylase has reduced activity, there is still detectable maltose generation, which can limit the growth of *Lactobacillus* at a sustained rate (Spear et al., 2015). Maltose is a single small-molecule compound maltooligosaccharide, which exists in women’s vagina and can be utilized by *Lactobacillus* (Ganzle and Follador, 2012). Maltose can be used to make maltose gel as a new non-antibiotic agent for the treatment of BV.

The vagina of female rhesus macaques (*Macaca mulatta*) is colonized by a group of anaerobic bacteria associated with BV in the woman (Chen et al., 2018). Rhesus macaque has been used as a BV animal model to study vaginal microbiota-associated diseases (Spear et al., 2010). Based on the results from the previous animal trials, the topical application of sucrose gel can induce the vaginal microbiota of rhesus macaques to change from BV-state microbiota to *Lactobacillus*-dominating microbiota (Hu et al., 2015). The current animal trial was designed to assess whether treatment with maltose gel is a better choice for BV than a placebo.

## Materials and Methods

### Animals and Sample Collection

Rhesus macaques, approximately 4 years of age, were provided by Sichuan Green-house Biotech Co., Ltd., and were deemed to be normal, healthy females at sample collection. Eighteen female rhesus macaques were randomly assigned, in a 2:1 ratio, to receive 0.5 g of maltose gel with maltose concentration of 2.0% (W/V) or 0.5 g of placebo gel by syringe to the fornix of the vagina for 5 consecutive days. The placebo formulation contained the same inactive matrix as maltose gel, which only lacked maltose compared with maltose gel. Both maltose gel (Palavigor Malt. Gel) and placebo gel are provided by Shenzhen Eulikan Biotechnology Co., Ltd. Sampling was scheduled on day 0, day 3, and day 5 during medication and on day 3 and day 5 after withdrawal (hereafter denoted as Day 0, Day 3, Day 5, After 3, and After 5, respectively). All animal studies were reviewed and approved by the Medical Ethics Committee of Beijing Tsinghua Changgung Hospital.

The vaginal discharge was obtained *via* two swabs. One swab was used to prepare a dry slide for Gram staining, under 1,000× magnification for visual detection. The other swab was quickly plunged into a tube containing 1 ml of PBS solution and stored at −80°C until the total DNA extraction of the vaginal microbiota.

### DNA Extraction, PCR Amplification, and Sequencing

The DNA of the sample was extracted through the TIANamp Bacteria DNA Kit (TIANGEN, China) according to the manufacturer’s instructions. This step required additional Lysozyme (Sigma-Aldrich), proteinase K, and RNase A (Sigma-Aldrich), and finally the DNA was washed and stored with 1 × TE buffer. A spectrophotometer was used (Thermo Scientific NanoDrop One) to measure the concentration and purity of the DNA extracts. Then, isolated DNA was stored at −80°C until needed.

16S rRNA was sequenced to determine the microbiota of samples. The V1–V2 regions of the 16S rRNA were amplified with universal primers 27F (5'-AGRGTTYGATYCTGGCTCAG-3') and 338R (5'-GCTGCCTCCCGTAGGAGT-3'). All PCR reactions were carried out with Phusion® High-Fidelity PCR MasterMix (New England Biolabs). The PCR products examined with 300–340 bp were chosen and mixed in equal density ratios. Then, the mixture PCR product was purified with the Qiagen Gel Extraction Kit (Qiagen, Germany). Sequencing libraries were generated using a TruSeq® DNA PCR-Free Sample Preparation Kit (Illumina, USA) following the manufacturer’s recommendations, and index codes were added. The library quality was assessed on the Qubit 2.0 Fluorometer (Thermo Scientific) and Agilent Bioanalyzer 2100 system. Last, the library was sequenced on an Illumina MiSeq platform.

### Sequencing Data Processing

The analysis of FASTQ files mainly adopted QIIME2 analysis suites (Bolyen et al., 2019). Firstly, sequencing reads from different samples were separated by duo barcodes with Cutadapt software (v2.8, https://cutadapt.readthedocs.io/en/stable/). Different FASTQ files for the same sample were gathered into one pair of FASTQ files. Then, all the single-sample FASTQ files were imported as QIIME2 artifacts. The raw sequences were denoised using DADA2 software wrapped in QIIME2 (--p-trunc-len-f 200 --p-trunc-len-r 200 --p-max-ee-f 4 --p-max-ee-r 4; Callahan et al., 2016). The denoised feature tables were filtered using QIIME2 feature-table filter-features (--p-min-frequency 10 --p-min-samples 2). The denoised sequences were classified using QIIME2 plugin feature-classifier classify-sklearn with trained Naive Bayes classifier on SILVA (132) taxonomic reference (Yilmaz et al., 2014; Bokulich et al., 2018). Finally, the phylogeny tree and alpha diversity were calculated from the filtered feature table. Further statistic tests and analyses were carried out using R.

### Nucleotide Sequence Availability

16S rRNA gene sequences in this study were deposited in the National Center for Biotechnology Information (NCBI) database on BioProject accession number: PRJNA645314.

## Results

### Diversity of Rhesus Macaque Vaginal Microbiota

Vaginal swabs were collected from 18 rhesus macaques (6 controls, numbered 1–6 and 12 in the test group, numbered 7–18). Due to menstruation and other uncontrollable factors, 8 rhesus macaques (1 control and 7 tests) all completed the collection of vaginal secretions at five time points during the follow-up period. Ten rhesus macaques (5 controls and 5 tests) missed some samples at different time points. We recorded the follow-up data of 18 macaques and collected 70 swab samples of vaginal secretions in two groups (see Supplementary Table 1).

**Figure 1:**
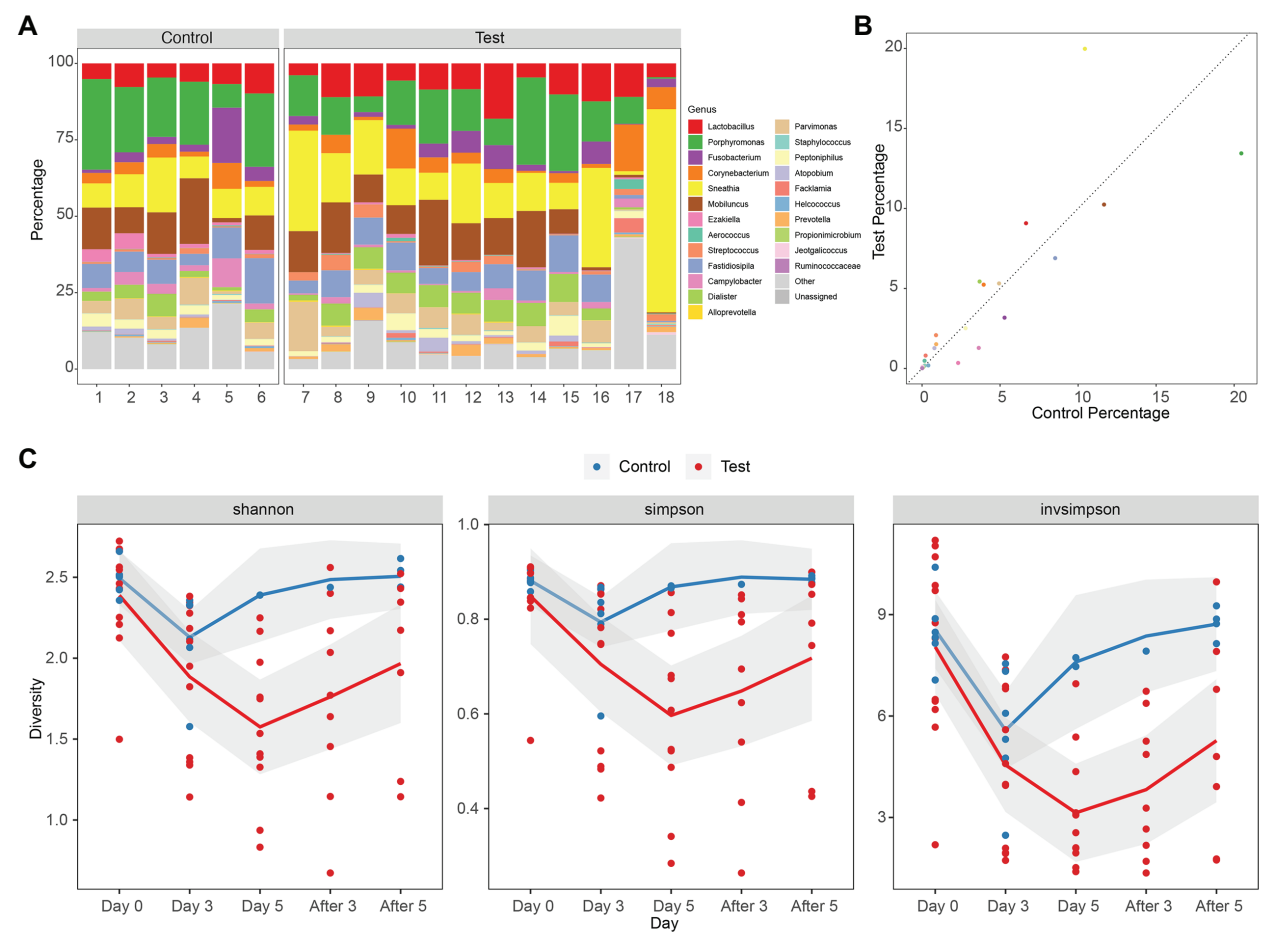
Diversity of rhesus macaque vaginal microbiota in control and test groups. **(A)** Composition of initial vaginal microbiota (Day 0). **(B)** The mean values of the primary genera of the initial vaginal microbiota (Day 0). **(C)** Alpha diversity of microbiota as measured using Shannon index, Simpson, and inverse Simpson of genus level 16S rRNA gene phylotypes. Control, blue; Test, red. The mean values of the five times of follow-up in two groups are connected by blue and red lines, respectively, and the gray area is the standard error of linear regression.

The vaginal microbiota of macaque was polymicrobial, and there was no significant difference between samples from control and test groups initially. The vaginal microbiota of macaques mainly included *Lactobacillus*, *Porphyromonas*, *Fusobacterium*, *Corynebacterium*, *Sneathia*, *Mobiluncus*, *Ezakiella*, *Aerococcus*, *Streptococcus*, *Fastidiosipila*, *Campylobacter*, *Dialister*, *Alloprevotella*, *Parvimonas*, *Staphylococcus*, *Peptoniphilus*, *Atopobium*, *Facklamia*, *Helcococcus*, *Prevotella*, *Propionimicrobium*, *Jeotgalicoccus*, and *Ruminococcaceae*, many of which are also found in women with BV (Figure 1A). There was no statistical difference of the mean values of the primary genera between the initial vaginal microbiota (Day 0) of control and test groups (two-tailed Student’s *t* test, *p* > 0.05), except for *Ezakiella* (*p* = 0.045 < 0.05) and *Streptococcus* (*p* = 0.038 < 0.05; Supplementary Table 2). However, when accounting for multiple tests, none of the 23 genera showed a significant difference between control and test groups (FDR > 0.05, Figure 1B; Supplementary Figure 1 and Supplementary Table 2).

Alpha diversity denotes the diversity of the ecosystem or microbiota. The diversity of vaginal microbiota of female rhesus macaques is similar to that of women with BV, which is higher than the diversity of vaginal microbiota of health women (Chen et al., 2018). Alpha diversities of the microbiota of different groups (Day 0, Day 3, Day 5, After 3, and After 5) were measured using the Shannon index, Simpson index, and inverse Simpson index (Invsimpson) from genus-level 16S rRNA gene phylotypes (Figure 1C; Supplementary Table 3). We did not find any significant difference (Shannon index *p* = 0.33 > 0.05, Simpson index *p* = 0.29 > 0.05, Invsimpson index *p* = 0.58 > 0.05) between control and test groups at Day 0 from the diversity indexes, which demonstrated that the initial microbiota diversities of samples from control and test groups were similar (Supplementary Tables 3 and 4). However, when we compared data from different days during treatment (Day 0, Day 3, and Day 5) in control and test groups, we could find that the diversity declined very significantly in test groups (ANOVA: Shannon index *p* = 0.00024 < 0.01, Simpson index *p* = 0.0021 < 0.01, Invsimpson index *p* = 0.000042 < 0.01), but not in control groups (ANOVA: Shannon index *p* = 0.036 > 0.01, Simpson index *p* = 0.12 > 0.01, Invsimpson index *p* = 0.015 > 0.01; Figure 1C). When it came to the days after treatment, we could observe an increasing trend in both control and test groups, although none of them were significant (ANOVA in control: Shannon index *p* = 0.28 > 0.05, Simpson index *p* = 0.052 > 0.05, Invsimpson index *p* = 0.067 > 0.05; ANOVA in test: Shannon index *p* = 0.34 > 0.05, Simpson index *p* = 0.47 > 0.05, Invsimpson index *p* = 0.17 > 0.05). These results indicated that the treatment can significantly reduce the diversity of vagina microbiota of female macaques. Although the diversity index of the treatment group increased after the withdrawal of medication, it remained at a lower level compared with the situation before medication. It indicated that the prebiotics still had a certain lasting effect on maintaining the stability of the bacterial community after drug withdrawal.

**Figure 2:**
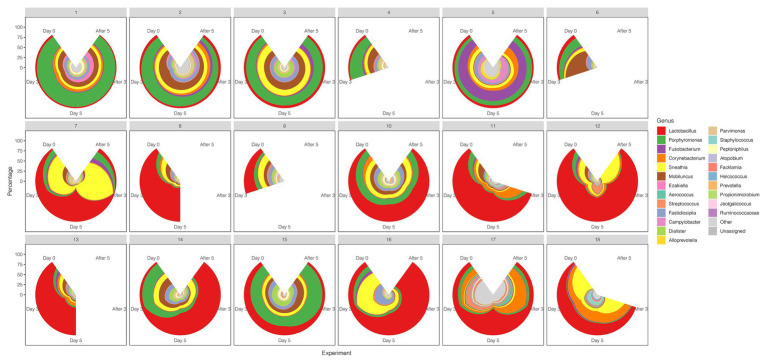
The diversity of the vaginal microbiota during the follow-up of each sample in control and test groups. The radius represents the percentage of the genus.

**Figure 3:**
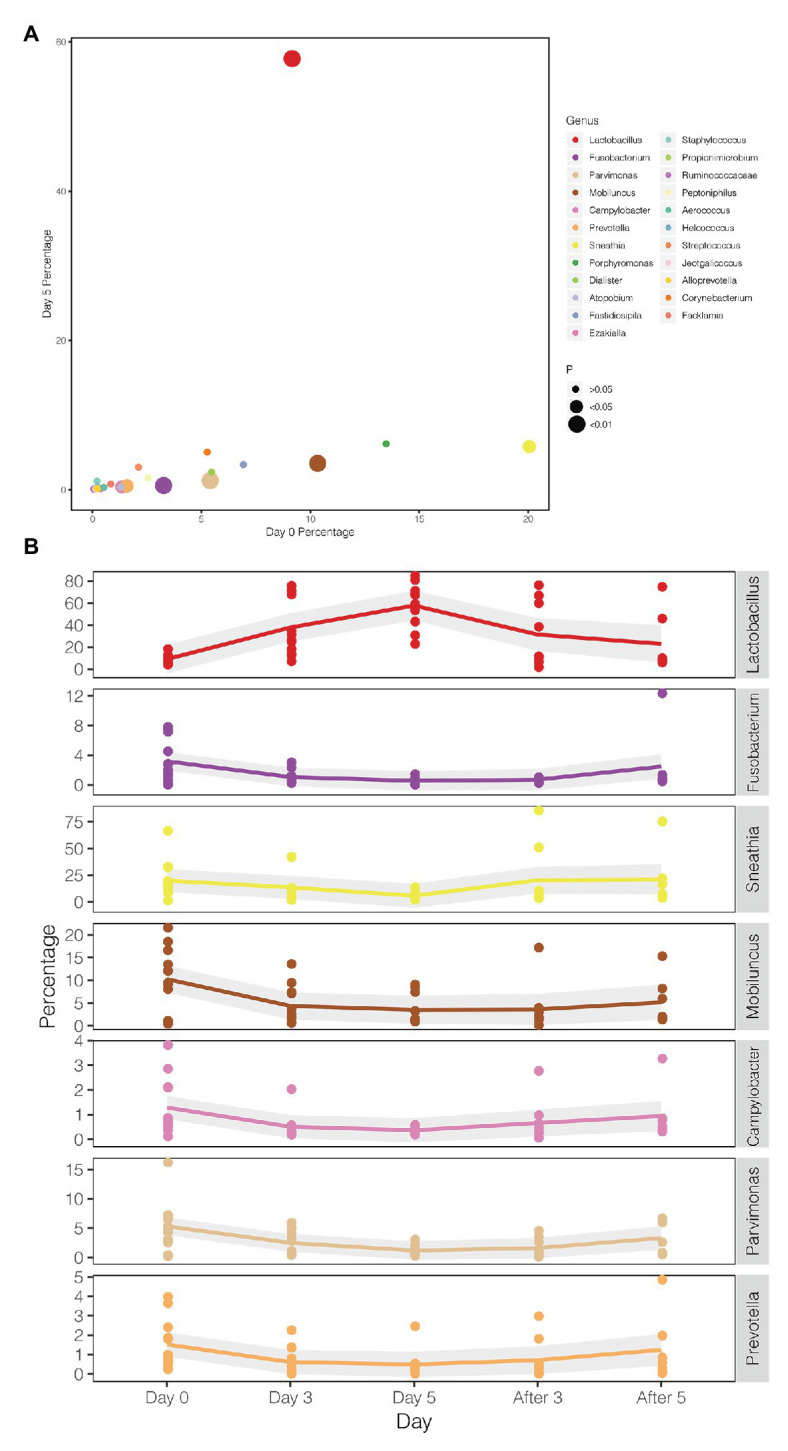
Changes in the abundance of bacteria with statistical difference in test group. **(A)** The percentage of bacteria on Day 0 and Day 5 in the test group. **(B)** The mean values of the five follow-up points are connected by different colored lines, respectively, and the gray area denotes the standard error.

A comparison between the test group and the control group showed that the maltose gel could promote the dominance of *Lactobacillus* in vagina microbiota. In the six samples of the control group (numbers 1–6), there was no significant change in the composition and diversity of vaginal microbiota from Day 0 to Day 5 (Supplementary Table 5), with the percentage of *Lactobacillus* declining slightly (Day 0 and Day 3, two-sided Student’s *t* test *p* = 0.016 > 0.1). In the test group (sample number 7–18), the abundance of *Lactobacillus* in the vagina microbiota increased gradually with the prolongation of the treatment time on Day 3 and Day 5 (ANOVA *p* = 6.99e−5 < 0.01; Supplementary Table 6). After drug withdrawal, the abundance of *Lactobacillus* decreased gradually and was higher than the initial vaginal microbiota on After 3 and After 5 (Figure 2). Based on the above results, the microbiota of the test group was greatly affected by maltose gel. We compared the relative abundance of bacteria on Day 0 and Day 5 in the test group. As shown in Figure 3A, the abundance of *Lactobacillus*, *Fusobacterium*, *Parvimonas*, and *Mobiluncus* had a significant statistical difference between Day 0 and Day 5 (ANOVA *p* < 0.01), and the abundance of *Campylobacter*, *Prevotella*, and *Sneathia* had a statistical difference (ANOVA *p* < 0.05; Supplementary Table 6). By evaluating the changing trend of the above genus at different time points, we found that the abundance of *Lactobacillus* increased from the beginning of the treatment to Day 5, but began to decrease after the withdrawal of maltose gel (Figure 3B). Correspondingly, the abundance of *Fusobacterium*, *Parvimonas*, *Mobiluncus*, *Campylobacter*, *Prevotella*, and *Sneathia* decreased on Day 0 to Day 5 and increased after drug withdrawal (Figure 3B).

**Figure 4:**
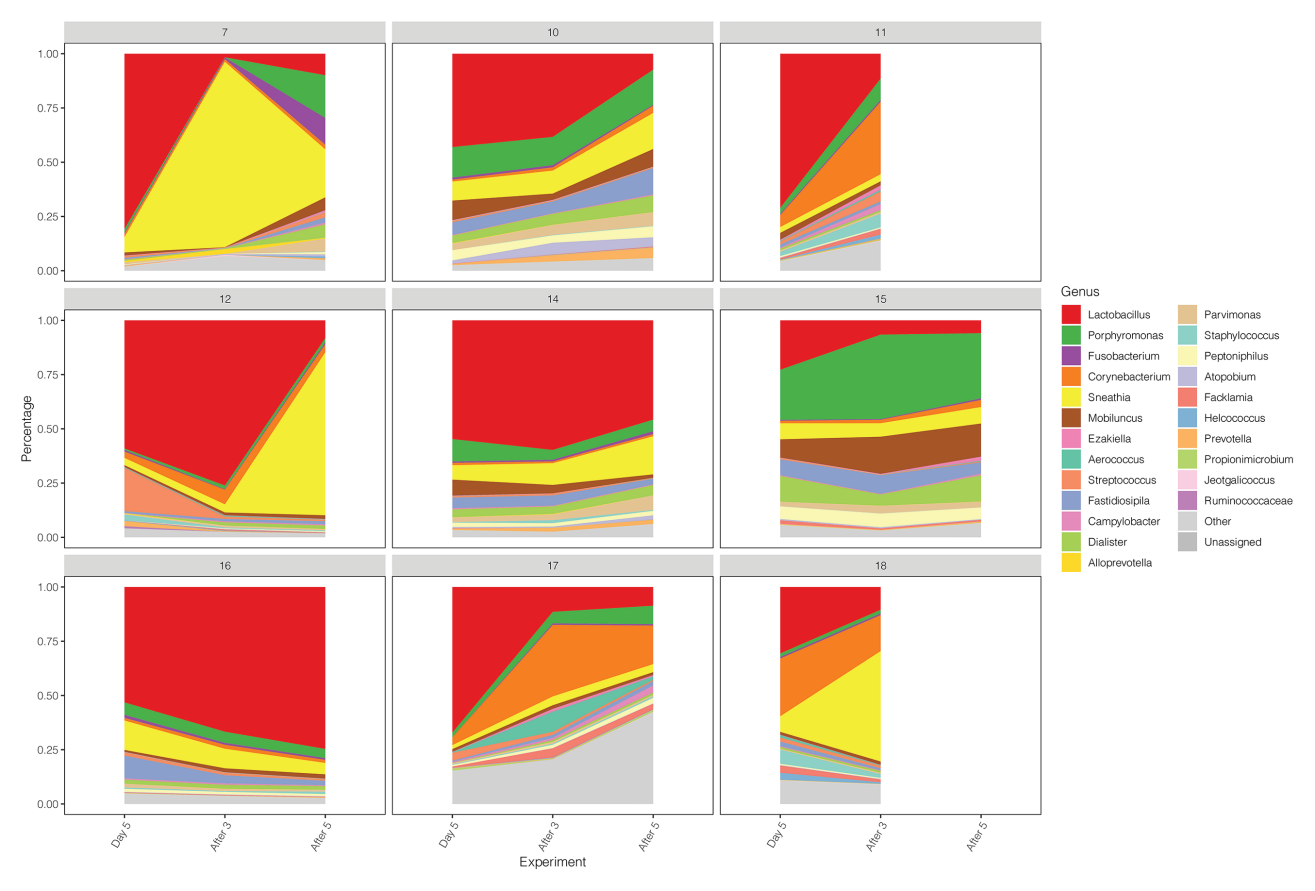
Diversity and abundance of vaginal microbiota between Day 5, After 3, and After 5 in test group.

Early drug withdrawal might lead to a mild relapse of the BV-like situation in the macaque vagina. When comparing Day 5 data with two follow-ups after withdrawal (After 3 and After 5), the percentage of *Lactobacillus* in most samples decreased gradually with the prolongation of withdrawal time, the dominant position of *Lactobacillus* was replaced, and the diversity of vaginal microbiota increased (Figures 1C, 4; Supplementary Table 7). However, the percentage reduction of *Lactobacillus* in the microbiota was moderate (ANOVA *p* = 0.016 > 0.01, Supplementary Table 7).

**Figure 5:**
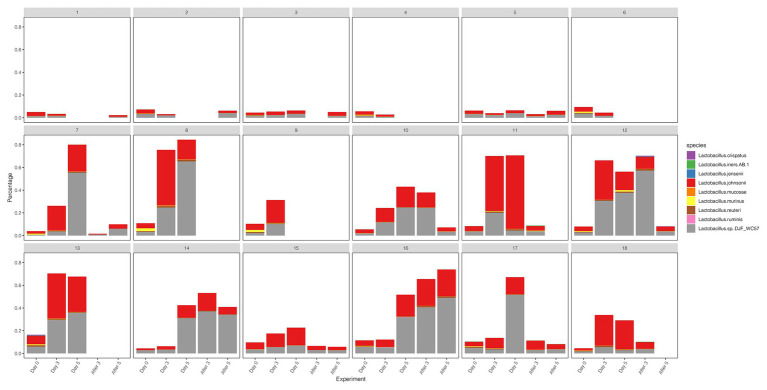
The diversity and abundance of the *Lactobacillus* during the follow-up of each sample in control and test groups.

We compared the diversity and abundance of *Lactobacillus* during follow-up between the control and test group. *Lactobacillus* sequences (mostly *L. johnsonii*) were found in rhesus macaques, and others include *L. crispatus*, *L. iners*, *L. jensenii*, *L. mucosae*, *L. murinus*, *L. reuteri*, *L. ruminis*, etc. (Figure 5; Supplementary Table 8). In the placebo gel control group, there was little change in the abundance of *Lactobacillus*. However, the abundance of *Lactobacillus* increased during the treatment period and gradually decreased after the withdrawal of the drug in the test group.

### Gram Stain of Vaginal Microbiota Before and After Gel Treatment

**Figure 6:**
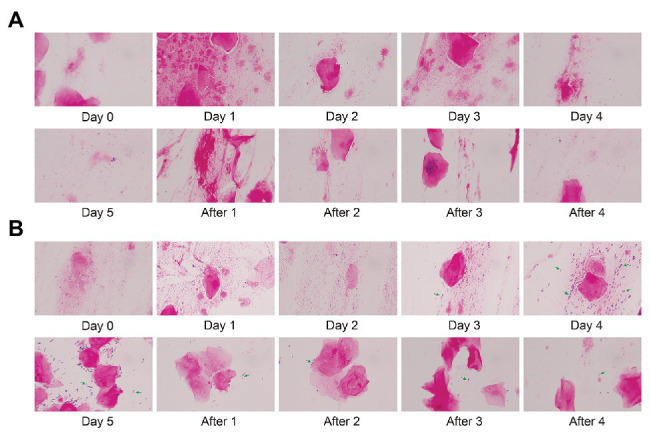
Gram stain of vaginal microbiota before and after gel treatment. **(A)** The morphology of Subject 5 (control group) under 1,000× magnification after gram staining during placebo gel treatment and withdrawal. **(B)** The morphology of Subject 16 (test group) during maltose gel treatment and withdrawal. Green arrows indicate *Lactobacillus*.

We stained the vaginal secretions with Gram obtained during different periods of follow-up. In this longitudinal analysis, we select respectively Subject 5 and Subject 16 in control and test groups to display a dynamic morphology under 1,000× magnification. There was almost no significant change in the morphology of Subject 5 before and after treatment (Figure 6A). The morphology of Subject 16 did not change significantly within 3 days after medication (Day 0, Day 1, and Day 2). In Figure 6B, *Lactobacillus* (green arrows) gradually appeared in the microscope field on Day 3 and Day 4, and the content of Day 4 increased compared with Day 3. Compared with Day 4, there were fewer cocci or vibrio in the microscopic field on Day 5 and only a large number of Gram-positive macrobacillus (mostly *Lactobacillus*). This indicates that *Lactobacillus* is predominant in the vagina. During a follow-up of 4 days after withdrawal (After 1, After 2, After 3, and After 4), the number of *Lactobacillus* decreased gradually.

## Discussion

### Diversity of Rhesus Macaque Vaginal Microbiota

This study suggests that the rhesus macaque genital microbiota was relatively polymicrobial, mainly including *Porphyromonas*, *Mobiluncus*, *Sneathia*, *Fastidiosipila*, *Prevotella*, *Atopobium*, *Lactobacillus*, etc. The microbial profile is characterized by relatively low levels of *Lactobacillus* and high numbers of Gram-negative bacteria. At the same time, we observed that the background bacteria of the initial vaginal sample (Day 0) after Gram staining consisted of a large number of dense Gram-negative microbacilli and clue cells with unclear boundaries under 1,000× magnification. These microbial features are consistent with previous reports in macaque vagina and similar to the characteristics of BV in women (Spear et al., 2010; Amaral et al., 2017). Considering the characteristics of low *Lactobacillus* and high BV-related bacteria in macaque vagina, macaque has been gradually used as animal models to study human BV in recent years (Spear et al., 2010; Amaral et al., 2017; Chen et al., 2018).

Healthy vaginal flora is composed of more than 90% *Lactobacillus* (mainly *L. crispatus*, *L. iners*, *L. gasseri*, or *L. jensenii*; Ravel et al., 2011). BV occurs when there is a shift in this flora to include a greater proportion of mixed anaerobic bacteria, such as the *Gardnerella*, *Prevotella*, and *Atopobium* species (Force et al., 2020). Although the rhesus macaque is one of the most extensively used nonhuman primate models for human diseases (Simmons, 2016), the main difference between the human BV state and macaque vagina is that the abundance of *Gardnerella vaginalis* (dominant bacteria) in the former is significantly higher than this in the latter. Additionally, the *Lactobacillus* (non-dominant bacteria) in the macaque vagina is mainly *L. johnsonii*, which is not consistent with the *Lactobacillus* (often *L. iners*) in BV. Therefore, the microbiota naturally present in primates share great similarities with human pathogens, but the distinction of the predominant microbiota between macaque and human highlights the need for cautious interpretation of the results of animal model studies (Chen et al., 2018).

### Prebiotic Maltose Gel Can Induce the Transition From Polymicrobial State of BV to *Lactobacillus* Dominant

Compared with Day 0, the alpha diversity index (Shannon index, Simpson index, and Invsimpson) declined very significantly in test groups on Day 3 and Day 5 during maltose gel therapy, and the abundance of *Lactobacillus* was significantly higher with the prolongation of the treatment time than the placebo gel group. At the same time, the abundance of several BV-related bacteria, *Fusobacterium*, *Parvimonas*, *Mobiluncus*, *Campylobacter*, *Prevotella*, and *Sneathia*, decreased on Day 0 to Day 5. The results indicate that prebiotic maltose gel can promote the growth of *Lactobacillus* and induce the polymicrobial state of BV to *Lactobacillus* dominant state, which results in the inhibition of BV bacteria in rhesus macaque vagina.

The composition of the human vaginal microbiota ranges from *Lactobacillus* dominant to a microbiota with diverse anaerobic bacteria, most evident with the common condition BV, which can be associated with a myriad of symptoms and adverse health outcomes (Falconi-McCahill, 2019). Furthermore, after treatment with an antibiotic agent, 20–75% of women have recurrent BV within 3 months (Bradshaw et al., 2006). Antibiotics are the first choice for the treatment of BV in the clinic (Sherrard et al., 2018; Cohen et al., 2020). However, while antibiotics inhibit BV-related bacteria, they may also eliminate *Lactobacillus* (Goldstein et al., 2015; Anisimova and Yarullina, 2019). This may be one reason for the high recurrence rate of BV. Vaginal *Lactobacillus* spp. provide broad-spectrum protection by producing lactic acid, bacteriocins, and biosurfactants, and by adherence to the mucosa that forms barriers against pathogenic infection (Kovachev, 2018). The presence of *Lactobacillus* is an important factor in the prevention of BV infection than antibiotics.

Prebiotics are chemically stable, which can significantly improve the growth and function of microbiota (Singh et al., 2017). The study confirms that maltose gel can facilitate the proliferation of *Lactobacillus* and promote the vaginal microbiota from BV-related bacteria dominant to *Lactobacillus* dominant in the rhesus macaque. Our results showed that once the prebiotic maltose gel was discontinued, the levels of *Lactobacillus* decreased gradually, accompanied by the increase of diversity of the vaginal microbiota. After all, the prebiotic maltose gel is a favorable carbon source for *Lactobacillus*. *Lactobacillus* abundance shall decline when carbon source supplies are reduced. In addition, the macaques had high levels of BV-related bacteria in their vagina, and low levels of *Lactobacillus* originally, which might be the “normal” state of the female macaques. When we artificially interfered with the vaginal microbiome of macaques with prebiotics, it led to the growth of *Lactobacillus* and the decline of BV-related bacteria. Once the intervention ceases, the physiological regulation of macaques may begin to play a role in promoting “health,” tending towards the initial bacterial state. The current trial evaluated 5 days of treatment and 5 days of follow-up; the further study should be considered to assess the longer-term sustainability of colonization and prevention of BV. Long-term use of maltose gel may maintain the dominance of *Lactobacillus*.

Glycogen in the vaginal epithelial cells is catabolized by human α-amylase to maltose, maltotriose, and α-dextrins, which are then metabolized to lactic acid by *Lactobacillus* species (Spear et al., 2014). This creates an acidic environment (pH, 3.5–4.5) conducive for the growth of *Lactobacillus* at the expense of other anaerobic bacterial species (Amabebe and Anumba, 2018). In our previous research, sucrose gel has a good performance in promoting the proliferation of *Lactobacillus* (Hu et al., 2015), but then we found that sucrose needs to be stable at around pH 5.5. When sucrose gel is used in the vagina, it will interfere with the normal pH of the vagina. However, maltose is more stable in an environment of pH 3.8, and as a decomposition product of glycogen under natural vagina environment, it is more suitable for selection as a vaginal prebiotic (Spear et al., 2015). Besides, unlike antibiotics, prebiotic substances rarely cause allergies (Pretorius et al., 2018) and do not provoke resistance. While prebiotics may be inferior to antibiotics against pathogens, the properties mentioned above make them a natural substitute for antibiotics (Markowiak and Slizewska, 2017). Based on the advantage above, prebiotic maltose gel may be possible to treat female BV clinically in the future. Notably, different prebiotics will stimulate the growth of different indigenous bacteria (Markowiak and Slizewska, 2017). *Lactobacillus* in the macaque reproductive tract is mainly *L. johnsonii*, and also *L. crispatus*, *L. iners*, *L. jensenii*, etc., which are common in the human vagina. Therefore, it may require *in vitro* and *in vivo* experiments to prove whether maltose gel is suitable for the treatment of human BV before it can be clinically applied in the future. One of the shortcomings of this study is that the number of macaques is a bit small. However, as our preliminary experiment, this study has confirmed that prebiotic maltose gel can promote the growth of *Lactobacillus* and induce a shift in the polymicrobial state of BV to *Lactobacillus* dominant. Then, we will expand the sample size for more detailed experimental verification in the future.

## Data Availability Statement

The datasets presented in this study can be found in online repositories. The names of the repository/repositories and accession number(s) can be found in the article/Supplementary Material.

## Ethics Statement

The animal study was reviewed and approved by Medical Ethics Committee of Beijing Tsinghua Changgung Hospital.

## Author Contributions

All authors were involved in the conception and design of the study. Q-qZ processed the samples and performed 16S rRNA gene sequencing. Q-qZ, Z-hL, and LZ analyzed the 16S rRNA data. L-lL, GH, and G-lL were responsible for maltose gel administration and follow-up in macaques. Q-qZ, Z-hL, and Q-pL contributed to study design and wrote the manuscript. Q-qZ and YW contributed to obtaining images of vaginal secretions after Gram staining. YC and WW helped coordinate and edit the manuscript. All authors reviewed and approved the final manuscript.

### Conflict of Interest

L-lL was employed by the company Shenzhen Eulikan Biotechnology Co., Ltd., China. GH and G-lL were employed by the company Sichuan Green-house Biotech Co., Ltd., China. YC and WW were employed by the company Suzhou Turing Microbial Technologies Co., Ltd., China.

The remaining authors declare that the research was conducted in the absence of any commercial or financial relationships that could be construed as a potential conflict of interest.
