# Supplementary figures and images for: Prebiotic Maltose Gel Can Promote the Vaginal Microbiota From BV-Related Bacteria Dominant to Lactobacillus in Rhesus Macaque
Source: Front Microbiol. 2020 Nov 6;11:594065. doi: 10.3389/fmicb.2020.594065 (PMC7677408; doi:10.3389/fmicb.2020.594065)

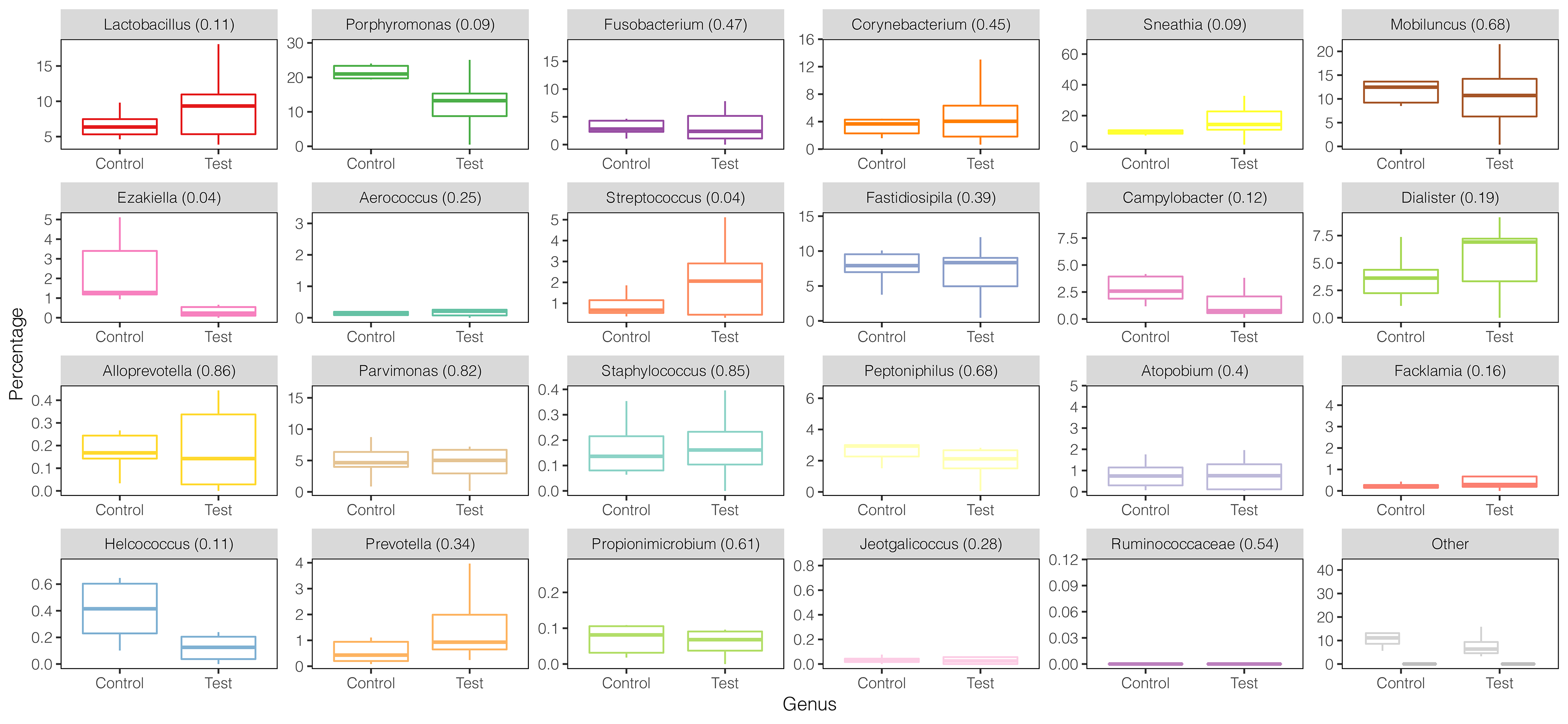

Supplement: Supplementary file 2 [file Image_1.tif]
